# Enhancement of the Piezocatalytic Response of La‐Doped BiFeO_3_ Nanoparticles by Defects Synergy

**DOI:** 10.1002/smll.202406425

**Published:** 2024-09-30

**Authors:** Wafa Amdouni, Mojca Otoničar, David Alamarguy, Emre Erdem, Pascale Gemeiner, Frédéric Mazaleyrat, Hager Maghraoui‐Meherzi, Jens Kreisel, Sebastjan Glinsek, Brahim Dkhil

**Affiliations:** ^1^ CentraleSupélec Laboratoire Structures Propriétés et Modélisation des Solides Université Paris‐Saclay UMR CNRS 8580 Gif‐sur‐Yvette 91190 France; ^2^ Faculté des Sciences de Tunis Laboratoire de Chimie Analytique et Électrochimie LR99ES15 Campus Universitaire de Tunis El‐Manar Université de Tunis El‐Manar Tunis 2092 Tunisie; ^3^ Jožef Stefan Institute Jamova 39 Ljubljana 1000 Slovenia; ^4^ CentraleSupélec CNRS Laboratoire de Génie Electrique et Electronique de Paris Université Paris‐Saclay Gif‐sur‐Yvette 91192 France; ^5^ Faculty of Engineering and Natural Sciences & Center of Excellence for Functional Surfaces and Interfaces for Nano‐Diagnostics (EFSUN) Sabanci University Orhanli Istanbul 34956 Turkey; ^6^ ENS Paris‐Saclay, CNRS, SATIE Université Paris‐Saclay Gif‐sur‐Yvette 91190 France; ^7^ Department of Physics and Materials Science University of Luxembourg Belvaux L‐4422 Luxembourg; ^8^ Luxembourg Institute of Science and Technology 41 rue du Brill Belvaux L‐4422 Luxembourg

**Keywords:** BiFeO_3_, defect dipoles, ferroelectric, oxygen vacancy, piezocatalysis

## Abstract

Because of their intrinsic polarization and related properties, ferroelectrics attract significant attention to address energy transformation and environmental protection. Here, by using trivalent‐ion‐lanthanum doping of BiFeO_3_ nanoparticles (NPs), it is shown that defects and piezoelectric potential are synergized to achieve a high piezocatalytic effect for decomposing the model Rhodamine B (RhB) pollutant, reaching a record‐high piezocatalytic rate of 21 360 L mol^−1^ min^−1^ (i.e., 100% RhB degradation within 20 min) that exceeds most state‐of‐the art ferroelectrics. The piezocatalytic Bi_0.99_La_0.01_FeO_3_ NPs are also demonstrated to be versatile toward various pharmaceutical pollutants with over 90% removal efficiency, making them extremely efficient piezocatalysts for water purification. It is also shown that 1% La‐doping introduces oxygen vacancies and Fe^2+^ defects. It is thus suggested that oxygen vacancies act as both active sites and charge providers, permitting more surface adsorption sites for the piezocatalysis process, and additional charges and better energy transfer between the NPs and surrounding molecules. Furthermore, the oxygen vacancies are proposed to couple to Fe^2+^ to form defect dipoles, which in turn introduces an internal field, resulting in more efficient charge de‐trapping and separation when added to the piezopotential. This synergistic mechanism is believed to provide a new perspective for designing future piezocatalysts with high performance.

## Graphical abstract

Defect and piezopotential synergy as a route for enhancing piezocatalytic performances: Piezoelectric La‐doped BiFeO_3_ nanoparticles are fabricated by low‐temperature chemical route, to investigate how point defects affect the piezocatalytic properties of BiFeO_3_. We show that the presence of oxygen vacancies and Fe^2+^providing further reactive sites, more carriers better charge transfer, and internal defect‐dipole fields leads to a record‐high piezocatalytic value in Bi_0.99_La_0.01_FeO_3_. The finding provides a new strategy for improving the piezocatalytic activity of BiFeO_3_.

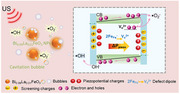

## 1 Introduction

Ferroelectric (FE) materials, such as the classical Pb(Ti,Zr)O_3_ (PZT), and BaTiO_3_ (BTO) perovskite oxides, possess a switchable spontaneous electric polarization under an external electric field. Because the polarization is also sensitive to mechanical stresses, temperature changes, or light excitation, FEs have been widely considered for non‐volatile memories^[^ ^1^ ^]^ or field‐effect transistors,^[^ ^2^ ^]^ sensors^[^ ^3^ ^]^ or actuators,^[^ ^4^ ^]^ coolers,^[^ ^5^ ^]^ and optical modulators,^[^ ^6^ ^]^ among others. In addition to these applications and due to their multisource energy harvesting ability,^[^ ^7^ ^]^ they are currently attracting a lot of attention in energy production, but are also widely considered to target environmental issues. For instance, harvesting mechanical vibration to convert it to electrical/chemical energy represents a new strategy to trigger chemical redox reactions, otherwise known as piezocatalysis. To be more specific, when mechanical force is applied to ferro/piezoelectric materials, a piezoelectric potential or piezopotential (i.e., built‐in electric field) is generated due to their non‐centrosymmetric structure, that drives charge transfer from the piezocatalysts to the surrounding medium to initialize chemical reactions via reactive oxygen species (ROS) generation.^[^ ^8^ ^]^ This emerging functionality has enabled broad engineering applications in pollutant degradation,^[^ ^8^, ^9^ ^]^ cancer and neurodegenerative therapy,^[^ ^10^ ^]^ H_2_ production,^[^ ^11^ ^]^ CO_2_ reduction,^[^ ^12^ ^]^ N_2_ fixation,^[^ ^13^ ^]^ and sterilization.^[^ ^14^ ^]^ Among these latter, pollutant degradation from wastewaters by utilizing mechanical energy lately received much more attention due to its prospect of solving increasingly severe environmental problems.

To achieve an excellent piezocatalytic performance, in our previous work we demonstrated that i) nanosized particles are required to limit the recombination of electrons (e^−^) and holes (h^+^), and favor their rapid migration to the particle surface to fully participate in the catalytic reactions, ii) a strong piezoelectric coefficient is needed despite the nanosizing which tends to suppress the ferroelectric/piezoelectric properties, iii) high polarization is demanded to favor e^−^‐h^+^ pairs separation and thus increase their lifetime by reducing their recombination, iv) low dielectric response is necessary to favor the piezoelectric figure of merit, as well as a high elastic modulus.^[^ ^8^ ^]^ Taken together, these unique properties make BiFeO_3_ (BFO) nanoparticles an ideal piezocatalyst for complex wastewater purification by harvesting mechanical energy. Furthermore, combined with light illumination, BFO is found to exhibit ultrahigh piezo‐photocatalytic efficiency allowing the degradation rate of Rhodamine B (RhB) dye to reach 41 750 L mol^−1^ min^−1^, which is much higher than that of other known FEs and the record value at that moment.^[^ ^8^ ^]^

Doping engineering at A‐ and/or B‐sites in ABO_3_ perovskite oxides has been demonstrated as one of the common approaches to not only enhance the photo‐induced response of the material in photocatalysis and related applications,^[^ ^1^, ^15^ ^]^ but also to modulate the density of defects, such as oxygen vacancies as it has been shown in, for example, ref. [16] in which oxygen vacancies controlled by aliovalent doping of Ca^2+^ at Bi^3+^ site is shown to influence the photoconductivity in BFO nanoparticles (NPs). In Pei et al work,^[^ ^17^ ^]^ by introducing Ca^2+^ and Mn^4+^ into BFO to modify the concentration of oxygen vacancies, the optical bandgap was significantly reduced to ≈2.41 eV and the remanent polarization was enhanced to ≈92.5 µC cm^−2^ leading, to an improvement of the photovoltaic (PV) effect. By using transition metal ions doping (such as Mn^2+^, Ni^2+^, Co^3+^, Cr^3+^, and Cu^2+^), Matsuo et al.^[^ ^18^ ^]^ showed that defect states within the bandgap (i.e., gap states), can be created and act as a scaffold for photogeneration, enabling a robust PV response. Moreover, Soltani et al.^[^ ^19^ ^]^ observed an enhancement by a factor of ≈1.5 of the visible light photocatalytic activity in Ba^2+^‐doped BFO NPs for benzene degradation. Such enhancement was attributed to the increased optical absorption, decreased oxygen vacancy content, and large surface area. Despite La^3+^ is not expected to affect the electroneutrality when substituting Bi^3+^ sites, similar observations were made on La‐doped BFO polycrystalline thin films.^[^ ^20^ ^]^ The authors of ref. [21] also found that La‐doping reduces the bandgap from 2.37 to 2.21 eV and ultimately enhances the photocatalytic efficiency of BFO. However, how defect concentration affects the piezocatalytic activity of BFO for wastewater treatments remains elusive. A recent work on Sm‐doped Pb(Mg_1/3_Nb_2/3_)O_3_‐PbTiO_3_ (Sm‐PMN‐PT) lead‐based system has proposed that the introduction of oxygen vacancies on the piezocatalyst surface promotes the adsorption of organic pollutants, resulting in higher degradation efficiency.^[^ ^22^ ^]^ Because the piezoelectric response of these piezocatalysts concomitantly decreases with the increase of the oxygen vacancy concentration, the piezodegradation reaches a maximum for an optimal oxygen vacancy amount. The same phenomenon was observed in BTO lead‐free ferroelectric materials.^[^ ^23^ ^]^ In contrast, a strong piezocatalytic activity in La‐doped BFO with up to 20% La concentration has been also recently suggested without mentioning any potential effects of defects.^[^ ^24^ ^]^ However, this piezocatalytic response raises questionable issues due to the high amount of parasitic phases that have been observed.

Here, for the first time, defects, not only oxygen vacancies as commonly reported,^[^ ^25^ ^]^ and piezopotential are synergized to achieve a giant piezocatalytic effect in modified prototypical multiferroic BFO. La‐doped BFO NPs were fabricated using a simple and eco‐friendly chemical route. Taking piezo‐degradation of RhB pollutant in water as the model reaction, a quantitative relationship between oxygen vacancies concentration and piezocatalytic properties is established. Intriguingly, we observe that Bi_0.99_La_0.01_FeO_3_ NPs display significantly enhanced piezocatalytic activity, as well as wide versatility enabling to piezo‐degrade various pharmaceuticals. When subjected to ultrasonic wave in the dark, the Bi_0.99_La_0.01_FeO_3_ NPs are found to have a record‐high piezocatalytic efficiency, as the piezocatalytic response dramatically improves allowing the degradation rate of the RhB dye to reach 21 360 L mol^−1^ min^−1^ (i.e., ≈100% degradation ratio within 20 min), which is the fastest degradation rate among the reported values and even surpassing that of previously reported piezocatalysts. Such remarkable efficiency is explained by the concomitant presence of oxygen vacancies and defect dipoles. The oxygen vacancies are proposed to provide further reactive sites, additional charges, and better charge transfer, while defects dipoles provide an internal electric field, resulting in efficient charge separation when coupled to the stress‐induced piezopotential.

## 2 Results and Discussion

### 2.1 Catalysts Characterization

**Figure 1:**
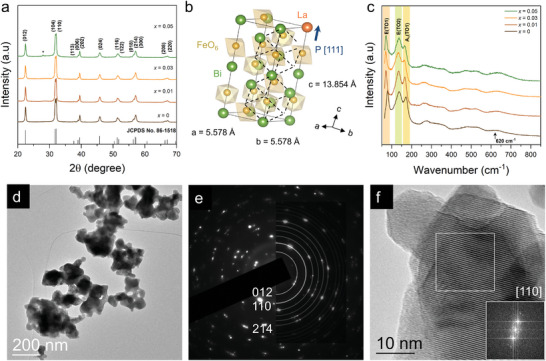
Structural and morphological characterizations. a) X‐ray diffraction patterns of Bi_1‐x_La_x_FeO_3_ nanoparticles. The peak marked with an asterisk corresponds to the Bi_2_Fe_4_O_9_ phase. b) Hexagonal crystal structure of La‐doped BiFeO_3_. Dashed lines represent the pseudo‐cubic unit cell. c) Room temperature Raman spectra of Bi_1‐x_La_x_FeO_3_ nanoparticles. d) TEM image of Bi_0.99_La_0.01_FeO_3_ nanoparticles and e) their corresponding SAED pattern with a simulated ring pattern superimposed. Indexing of the SAED pattern was done according to the R3c rhombohedral symmetry. f) High‐resolution TEM image of Bi_0.99_La_0.01_FeO_3_ nanoparticles with insets showing the FFT pattern of the white‐squared region.

The crystal structure and phase purity of Bi_1‐x_La_x_FeO_3_ (BLFO) NPs with the nominal concentrations *x* = 0, 0.01, 0.03, and 0.05 synthesized via the low‐temperature chemical bath method, were first investigated by X‐ray diffraction (XRD) as shown in **Figure** **1**a, where all the diffraction peaks can be well indexed as rhombohedral distorted perovskite structure with space group *R3c* (JCPDS card No. 86–1518), as illustrated in Figure 1b using a hexagonal setting. In contrast to other works,^[^ ^24^ ^]^ no peaks corresponding to any impurity or secondary phases were detected, except for *x* = 0.05, where traces of the mullite Bi_2_Fe_4_O_9_ phase are observed. The structure parameters extracted from refinement (Figure S1, Supporting Information) are summarized in Table S1 (Supporting Information). The structure of BLFO NPs was also studied using Raman spectroscopy at room temperature, as shown in Figure 1c. All the 13 Raman active modes (4A_1_+9E) corresponding to the *R3c* symmetry of BFO are observed in BLFO NPs, as predicted by group theory.^[^ ^26^ ^]^ No other Raman mode derived from impurity phases was detected, further confirming that all Bi_1‐x_La_x_FeO_3_ (0 ≤ *x* ≤ 0.05) NPs keep the same space group as BFO, which is consistent with XRD results. To obtain the exact peak position, each measured Raman spectrum of BLFO NPs was fitted using a deconvolution into individual Lorentzian components and the corresponding fitting results are given in Table S2 (Supporting Information). The mode assignment is in agreement with those previously reported in the literature,^[^ ^8^, ^27^ ^]^ the Bi atoms participates in low wavenumber vibration modes below 170 cm^−1^, oxygen motions strongly determine the modes above 264 cm^−1^, and Fe motions are mainly involved in the modes between 152 and 261 cm^−1^. We note that only the intensity and the position of the modes are affected by La‐doping. Indeed, at low wavenumber range (Figure 1b; magnified in Figure S2a, Supporting Information), it can be seen that the E(TO1) at ≈79 cm^−1^ (orange shaded area), E(TO2) at ≈140 cm^−1^ (green shaded area) and A_1_(TO1) at ≈170 cm^−1^ (yellow shaded area) modes shift toward lower wavenumbers with La‐doping. While La has a lighter atomic mass compared to that of Bi, this observation implies a change of the Bi─O covalent bonds as a result of the decline in the stereochemical activity of the Bi electronic lone pair (arising from hybridization between 6s and 6p orbitals). For instance, the E(TO1), whose atomic motions can be described as an antiphase vibration between the Bi‐site and FeO_6_ sublattices, downshift by ≈10 cm^−1^. Note that there is a notable change of behavior (Figure S2a, Supporting Information) for BLFO NPs with *x* = 0.05, which likely reflects the consequence of the presence of parasitic phases evidenced using XRD. We also observed a redshift on the Fe‐site, such as on the E mode at ≈260 cm^−1^ associated to the Fe─O covalent bond and corresponding Fe─O─Fe angle,^[^ ^27^, ^28^ ^]^ which can affect the antiferromagnetic order. At high wavenumber range, the relative intensity of the ≈620 cm^−1^ mode (Figure 1c), associated to silent A_2_ longitudinal optical mode activated by Fröhlich interactions^[^ ^28a^ ^]^ due to charge carriers‐phonon coupling, increases with La‐doping, when compared to BFO (see also Figures S2b and S3, Supporting Information). This might suggest that the number of polarons increases possibly leading to more local charge‐induced polarization and larger polarons allowing hopping of the self‐trapped charges.

The morphology of the as‐synthesized samples is analyzed by transmission electron microscopy (TEM). Pure BFO NPs have been reported to exhibit semi‐hexagonal to spherical morphology.^[^ ^8^, ^27^ ^]^ The morphology of Bi_0.99_La_0.01_FeO_3_ is hexagonal with semi‐faced polyhedral (Figure 1d) with an average size of ≈50 nm. Figure 1e shows a selected area electron diffraction (SAED) pattern of multiple NPs from Figure 1d and a superimposed ring pattern simulated from JCPDS card No. 86–1518, allowing to identify the perovskite reflections. The clear spots and no diffuse rings in SAED indicate the high crystallinity of the NPs. This is further confirmed by the clear crystal lattice fringes in the high‐resolution TEM image (Figure 1f) and the corresponding Fast Fourier Transform (FFT) (see inset). For BLFO NPs with *x* > 0.01, a round‐like morphology is observed (Figure S4, Supporting Information).

### 2.2 Piezocatalytic Performance

**Figure 2:**
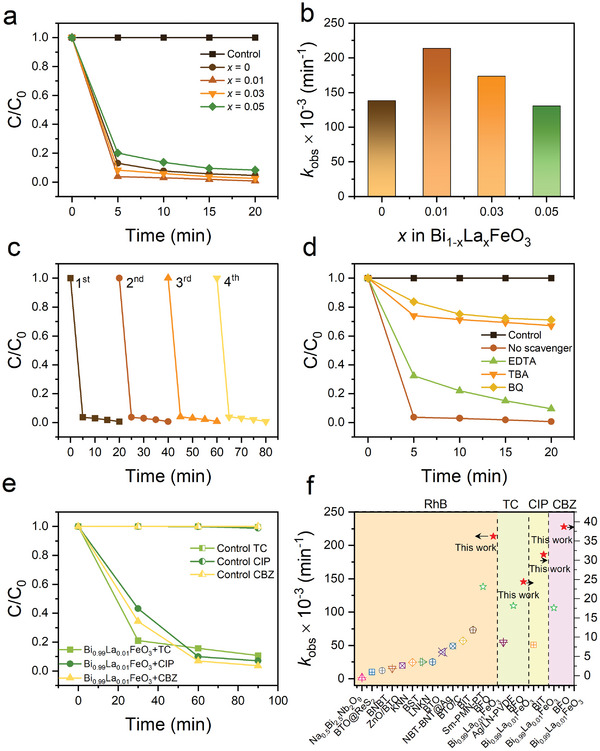
Piezocatalytic performance of Bi_1‐x_La_x_FeO_3_ (x = 0, 0.01, 0.03, and 0.05) nanoparticles. a) Degradation efficiency of RhB using Bi_1‐x_La_x_FeO_3_ in the dark under an ultrasonic wave (100 W and 45 kHz). b) The corresponding reaction k_obs_ constant. c) Recycling performance of Bi_0.99_La_0.01_FeO_3_ nanoparticles for RhB degradation. d) Piezo‐degradation of RhB with and without free radical scavengers in the presence of Bi_0.99_La_0.01_FeO_3_ nanoparticles. e) Piezo‐degradation efficiency of TC, CIP, and CBZ using Bi_0.99_La_0.01_FeO_3_ as catalysts. f) Reaction rate constant k_obs_ of BFO (green stars) and Bi_0.99_La_0.01_FeO_3_ (red stars) compared with other piezoelectrics materials (see details in Table S3, Supporting Information).

The degradation of Rhodamine B (RhB) is selected as a model reaction to evaluate the efficiency of the synthesized BLFO NPs for removing organic pollutants from water by the piezoelectric effect. Ultrasonication is used to simulate the mechanical stimuli and the temperature of water in the ultrasonic bath was maintained at room temperature (≈22 ± 2 °C) during the catalytic process, to avoid the effect of pyroelectric catalysis by thermal variation. At specific time intervals, ≈3 mL of filtered solution was collected for analysis. Corresponding photos and UV–vis absorption spectra are displayed in Figure S5 (Supporting Information). The intensity of the absorbance peak of RhB significantly decreased with increasing ultrasonication time, indicating the degradation of RhB molecules. From **Figure** **2**a, it can be clearly seen that Bi_0.99_La_0.01_FeO_3_ exhibits the highest degradation efficiency (brown upward triangles), and the piezo‐degradation follows the order of Bi_0.99_La_0.01_FeO_3_ > Bi_0.97_La_0.03_FeO_3_ > BiFeO_3_ > Bi_0.95_La_0.05_FeO_3_. The degradation ratio of RhB with Bi_0.99_La_0.01_FeO_3_ NPs reaches 96% within 5 min in the dark. When increasing the time to 20 min, the RhB is completely decomposed to reach 100%. In contrast, one can observe that there is no degradation in the absence of any one of the synthesized BLFO catalysts (Control in Figure 2a). Using pseudo‐first‐order reaction, as expressed by Equation (1):

(1) $$\ln\left(\frac{C_{0}}{C}\right)=k_{\mathrm{obs}}\;t$$

where C_0_ and C are the concentrations of RhB at time *t* = 0 and *t*, respectively. Note that the error bar on the *k* values is less than 10% including the fit and data acquisition errors. The observed rate constant (*k*_obs_) is found to be 0.2136 min^−1^ for Bi_0.99_La_0.01_FeO_3_ NPs (Figure 2b) which is, to the best of our knowledge, the highest degradation rate ever reported (see Table S3, Supporting Information). Indeed, this value is ≈133, 18 and 3 times higher than that of previously reported relaxor‐based ferroelectric, that is, Na_0.5_Bi_2.5_Nb_2_O_9_ (*k*_obs_ = 0.0016 min^−1^), 0.93(Bi_0.5_Na_0.5_)TiO_3_‐0.07BaTiO_3_ (BNBT) (*k*_obs_ = 0.012 min^−1^) and Sm‐PMN‐PT (*k*_obs_ = 0.073 min^−1^),^[^ ^22^, ^29^ ^]^ ≈11 and 9 times higher than that of perovskite‐based ferroelectrics, K_0.5_Na_0.5_NbO_3_ (KNN) (*k*_obs_ = 0.0198 min^−1^) and Ba_0.75_Sr_0.25_TiO_3_ (BSTO) (*k*_obs_ = 0.0245 min^−1^),^[^ ^30^ ^]^ ≈21 and 14 times higher than that of ferroelectric heterostructures BTO@ReS_2_ (*k*_obs_ = 0.01min^−1^) and ZnO/BTO (*k*_obs_ = 0.0153 min^−1^),^[^ ^11^, ^31^ ^]^ and ≈4 times higher than that of ferroelectric Bi_4_Ti_3_O_12_ (BIT) (*k*_obs_ = 0.057min^−1^) with Aurivillius structure (see Table S3, Supporting Information; Figure 2f).^[^ ^32^ ^]^ The specific rate constant *k* was further calculated by *k*_obs_/[M] with [M] being the concentration of RhB dye. The piezocatalytic rate constant is thus calculated to be 21 360 L mol^−1^ min^−1^, which is ≈1.54 times higher than that of pure BFO (*k* = 13 810 L mol^−1^ min^−1^).^[^ ^8^ ^]^ To our knowledge, this value is also ≈3 times much higher than that of recently reported graphene‐oxide/MoS_2_ heterostructure (*k* = 8400 L mol^−1^ min^−1^).^[^ ^9b^ ^]^

To further figure out the performance of such a BFO‐based catalyst, four consecutive cycles under a total of 80 min of ultrasonic excitation are conducted in the dark and show that our Bi_0.99_La_0.01_FeO_3_ NPs still retained the ultrahigh RhB degradation rate (Figure 2c; Figure S6a, Supporting Information). Besides, the crystalline structure of the nanocatalyst was not changed after reaction, indicating their high stability and good durability (Figure S6b, Supporting Information). To identify the reactive active species generated during the piezocatalytic process and their contribution, a series of quenching experiments were performed by introducing tert‐butyl alcohol (TBA), benzoquinone (BQ), and disodium ethylene diamine tetra‐acetate dehydrate (EDTA‐2Na) as scavengers for hydroxyl radicals (•OH), superoxide radicals (•O_2_^−^) and holes (h^+^), respectively, into the piezocatalytic system. As shown in Figure 2d and Figure S7a,b (Supporting Information), the piezocatalytic effect was significantly inhibited upon the addition of TBA or BQ, where the RhB degradation efficiency decreased from 100% by 67 and 71%, respectively, indicating that •OH and •O_2_^−^ radicals are of importance during the piezocatalytic degradation reaction. Meanwhile, after adding the hole scavenger EDTA‐2Na, the RhB removal ratio was slightly reduced by ≈10%, indicating that h^+^ is less important for direct piezodegradation and rather useful for producing •OH from h^+^ + OH^−^ → •OH. These results imply that •OH and •O_2_^−^ are the main ROS responsible for piezocatalytic degradation of RhB using Bi_0.99_La_0.01_FeO_3_ NPs, where their contributions are calculated to be ≈92% (see Figure S7, Supporting Information).

The accumulation of antibiotics and antibiotic resistance genes (ARGs) in water bodies presents a serious problem, potentially causing drug‐resistant bacteria and organ injury due to their toxicity and resistance to degradation. Therefore, to go beyond RhB, tetracycline (TC), ciprofloxacin (CIP), and carbamazepine (CBZ) were selected as three kinds of commonly used pharmaceuticals to reveal the versatility of our BLFO nanocatalysts. Figure 2e shows that the Bi_0.99_La_0.01_FeO_3_ nanocatalyst is able to piezo‐degrade all pharmaceutical pollutants, with over 90% removal efficiency within 90 min under ultrasound excitation. The corresponding *k*_obs_ values are presented in Figure S8 (Supporting Information) (see also Figure 2f; Table S3, Supporting Information). These results not only confirm that our BFO‐based catalyst can be used to simultaneously target a wide variety of organic pollutants,^[^ ^8^ ^]^ but also highlights the advantage of using piezocatalysis over conventional processes for pharmaceutical removal in waste water treatment plants, which are known to be insufficient in removing these micropollutants, either partially or totally. Moreover, one can see that 1% La‐doping of BFO is enough to permit a significant improvement of the piezocatalytic efficiency of our BFO,^[^ ^8^ ^]^ also for the degradation of TC, CIP, and CBZ with better performances than those reported in the literature (Figure 2f; Table S3, Supporting Information). These findings provide solid evidence that BLFO‐based piezocatalysts are very promising for wastewater treatment and could be naturally envisioned for other applications, including H_2_ production, hydrogen peroxide (H_2_O_2_) generation, and CO_2_ conversion or antibacterial and tumor therapy.

### 2.3 Synergistic Mechanism of Defects and Piezopotential

**Figure 3:**
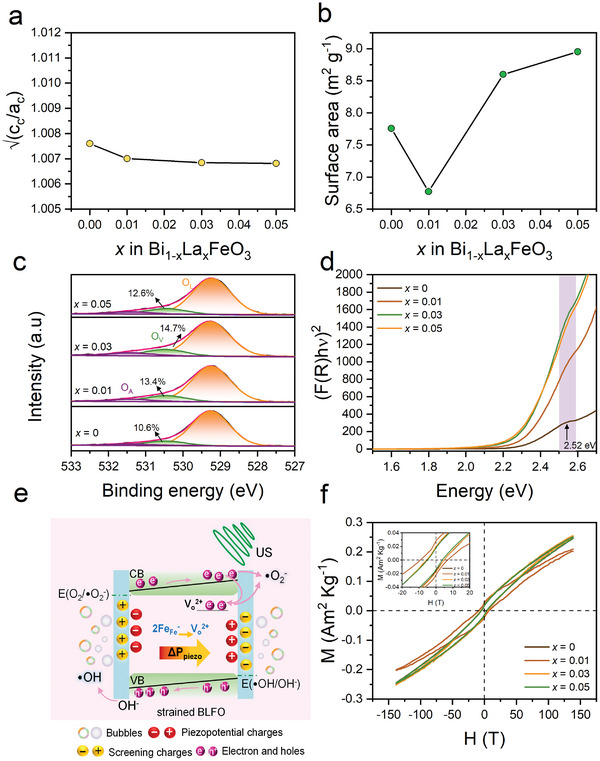
a) Composition dependence of the square root of tetragonality of Bi_1‐x_La_x_FeO_3_ nanoparticles. b) The surface area trend in Bi_1‐x_La_x_FeO_3_ nanoparticles. c) XPS high‐resolution spectra of O 1s of Bi_1‐x_La_x_FeO_3_ nanoparticles. d) Tauc plots of Bi_1‐x_La_x_FeO_3_ nanoparticles. e) Schematic of the mechanism of RhB enhanced degradation in Bi_0.99_La_0.01_FeO_3_ nanoparticles mediated piezocatalytic effect. f) The M‐H loops of Bi_1‐x_La_x_FeO_3_ nanoparticles. The inset is a zoom of the magnetization curves.

Now that we have shown the piezocatalytic performance of our BLFO NPs, the next question was to understand how and why Bi_0.99_La_0.01_FeO_3_ NPs exhibit the highest piezocatalytic response. Based on the literature,^[^ ^33^ ^]^ 1% La‐doping will only slightly, if any, impact the structure and polarization of BFO. In our previous work,^[^ ^8^ ^]^ we also highlight the difficulty and the many sources of measurement errors to assess the ferroelectric properties of NPs, and we could extract the piezoelectric coefficient of BFO NPs (that is, 64 ± 10 pm V^−1^) by combining studies on a macroscopic and nanoscopic scale with a comparative analysis between the responses of both the BFO NPs and the polymer in which the latter were embedded. Here, we have rather considered a proxy of the polarization by taking into account the classical relationship coupling the polarization to the strain in FEs. It is known that the distortion of the unit cell with respect to the cubic ideal lattice (c/a in case of tetragonal distortion) is proportional to the square of polarization (*P*), as given by Equation (2):^[^ ^34^ ^]^

(2) $$\frac{c}{a}\propto \;QP^{2}\;\mathrm{and}\;\mathrm{thus}\;P\propto \;\sqrt{\frac{c}{a}}$$

where *Q* is the electrostrictive coefficient and *P* = *P*_s_ is the spontaneous polarization. **Figure** **3**a shows the composition dependence of the proxy of the polarization calculated by the square root of the distortion represented by the c_pc_/a_pc_ ratio in the pseudo‐cubic notation calculated from corresponding lattice parameters *a*_pc_ and *c*_pc_ (see Table S1, Supporting Information) and assuming the electrostrictive coefficient is unchanged. As a result, when increasing La‐doping, the values of the polarization proxy are weakly changed and remain close to BFO, meaning that, as anticipated,^[^ ^33^ ^]^ the polarization is only slightly, if any at all, altered with so small La‐doping. Moreover, expanding the expression of the polarization as a function of field in Equation (2) leads to Equation (3):

(3) $$\frac{c}{a}\propto Q\;{\left(P_{s}+P_{i}\right)}^{2}=\;QP_{s}^{2}+2QP_{s}\varepsilon_{0}\varepsilon E+{\left(\varepsilon_{0}\varepsilon E\right)}^{2}$$

where *P*_i_ is the polarization induced by an electric field *E* (*P*_i_ = ε_0_ ε *E*, with ε_0_ the permittivity of vacuum and ε the dielectric constant). In Equation (3), the second term, linear with the electric field, corresponds to the piezoelectric coefficient *d = 2QP*_s_ ε_0_ ε.^[^ ^34^ ^]^ Because the dielectric constant does not change with so weak La‐doping,^[^ ^33^ ^]^ the piezoelectric coefficient, like the polarization, is slightly affected and cannot be considered as a key parameter that could here explain the piezocatalytic difference between samples. In catalysis, the surface area is believed to play a crucial role, as a material with a large surface area provides more active reaction and adsorption sites on the surface for the catalytic process, which leads to enhanced catalytic activity.^[^ ^35^ ^]^ Accordingly, to clarify the effect of surface area on the piezocatalytic activity of BLFO NPs, we carried out nitrogen (N_2_) adsorption measurements, and the corresponding results of BET (Brunauer‐Emmett‐Teller) surface area (S_BET_) are displayed in Figure 3b (see also Figure S9, Supporting Information; **Table** **1**). As one can see, while they possess lower piezocatalytic activity in comparison to Bi_0.99_La_0.01_FeO_3_ NPs (see Figure 2a,b), the BET surface area of BLFO NPs with *x* = 0.03 and 0.05 is higher than that of Bi_0.99_La_0.01_FeO_3_ NPs (S_BET_ = 6.8 m^2^ g^−1^). The S_BET_ in this doping regime (*x* ≥ 0.01) is in line with the particle size of the NPs (see Table 1), where the smaller the particles, the higher the surface area. The 60 nm‐sized pristine BFO exhibits a surface area of S_BET_ = 7.8 m^2^ g^−1^, which is ≈1.14 higher than that of Bi_0.99_La_0.01_FeO_3_ NPs with 53 nm average size. Such difference in BET surface area can be attributed to the morphological changes of the NPs (see Figure 1d,f and ref. [8]), allowing us to rule out the possibility that the material‐specific surface area is the main reason for the observed ultra‐fast piezocatalytic efficiency.

**Table 1:** Morphology, particle size, surface area, and observed rate constant *k*_obs_ for RhB degradation.

	Morphology	Average particle size [nm]	S_BET_ [m^2^ g^−1^]	*k*_obs_ constant [×10^−3^ min^−1^]
BiFeO_3_	Semi‐hexagonal to spherical	60	7.8	138.1
Bi_0.99_La_0.01_FeO_3_	Hexagonal	50	6.8	213.6
Bi_0.97_La_0.03_FeO_3_	Round‐like	38	8.6	173.6
Bi_0.95_La_0.05_FeO_3_	Round‐like	37	8.9	130.6

It is also widely recognized that oxygen vacancies are one of the most common point defects in FE oxides that can strongly affect their physico‐chemical properties. While substituting Bi^3+^ with La^3+^ should not, in principle, affect the stoichiometry and defect state, the aforementioned literature shows that in practice it does. We used X‐ray photoelectron spectroscopy (XPS) to investigate the composition and chemical states of related elements. The overview XPS survey and the core level spectra of Bi 4f, Fe 2p_3/2,_ and La 3d are provided in Figure S10 (Supporting Information). Figure 3c displays the O 1s XPS core spectra, which can be deconvoluted into three peaks at 529.22, 530.14, and 531.19 eV corresponding to lattice oxygen (O_L_), oxygen vacancies (O_V_), and surface adsorbed oxygen (O_A_), respectively. Notably, as clearly presented in the figure, the concentration of oxygen vacancies gradually increases from 10.6 to 14.7% with increasing La‐doping from *x* = 0 to 0.03 and then decreases for BLFO NPs with *x* = 0.05. Note that as already mentioned, BLFO with *x* = 0.05 shows parasitic phases that also contribute to the spectra and could then alter the chemical analysis, and thus here we do not consider this sample. The increase of oxygen vacancies with La doping is also supported using Electron Paramagnetic Resonance (EPR) measurements (Figure S11, Supporting Information). Indeed, the change of the lineshape of the EPR spectra due to additional contribution to the signal at a g‐factor of 4.2, and the shift in the g‐factor from 2.0159 to 2.0021 is explained by an increase in the concentration of oxygen vacancies as the La doping level increases. Interestingly, this latter shift suggests that oxygen vacancies in La‐doped BFO are predominantly located on the surface. As already reported,^[^ ^22^, ^23^ ^]^ the oxygen vacancies can improve the adsorption of reactant molecules at the surface of the catalysts, resulting in higher degradation efficiency. It has been also proposed that it exists an optimal oxygen vacancy amount because the oxygen vacancies are believed to reduce the piezoelectric response. Here as well, the piezo‐degradation reaches a maximum for Bi_0.99_La_0.01_FeO_3_ NPs while BLFO with *x* = 0.03 displays a higher oxygen vacancy amount. However, as discussed above, the change of the piezoelectric properties of our BLFO NPs between *x* = 0.01 and *x* = 0.03 is negligible, and could not alone explain our observations. Here, we would like to discuss other possible roles of these oxygen vacancies in the piezocatalytic response. Actually, it is well known^[^ ^36^ ^]^ that oxygen vacancies will introduce defects states in the band structure of BFO as depicted in the inset of Figure 3d. These defect states impact the absorption spectrum by reducing the optical band gap (2.6–2.9 eV)^[^ ^37^ ^]^ allowing the onset of absorption to vary from 2.29, 2.26, 2.22, and 2.19 eV for BLFO NPs with *x* = 0, 0.01, 0.03, and 0.05, respectively, when extracted using Tauc plot (see Figure S13, Supporting Information) from the light absorption measured by UV–vis diffuse reflectance spectroscopy (DRS); see Figure 3d. This apparent decrease of the absorption onset is mainly due to the increased oxygen vacancy concentration; however, in the case of BLFO NPs with *x* = 0.05, we cannot rule out the possible contribution of parasitic phase Bi_2_Fe_4_O_9_ to the absorption. The increase of the intensity of absorption at ≈2.5 eV (corresponding to oxygen vacancies energy level; the purple shaded area in Figure 3d) with La amount is in line with the increase of oxygen vacancies from XPS and EPR data. It is worth mentioning here that the creation of oxygen vacancy, which is positively charged $\;(V_{O}^{2+})$, involves the creation of two electrons to compensate for the total charge. These electrons can be self‐trapped by the oxygen defects, which could suggest the observation of the Raman mode at 620 cm^−1^ (see Figure 1c). It is also worth to note that the energy level of the oxygen defects is just below the conduction band and very close to the energy corresponding to the production of •O_2_ superoxide radical used for the piezocatalysis as illustrated in Figure 3e. As a result, when the BLFO NPs are subjected to ultrasound excitations, the stress‐induced piezopotential, which produces polarization charges at the surface to piezo‐degrade the pollutant molecules, will also provide an internal electric field which may participate to the piezocatalytic process by de‐trapping the electrons of the oxygen defects which energy level will favor the generation of ROS. Thus, the oxygen vacancies not only act as exciton trapping center that provides more adsorption surface sites beneficial for the piezocatalysis process, but also provides additional charges (transported by the hoping mechanism) and a better energy transfer between the NPs and surrounding molecules. Increasing the amount of oxygen vacancies, however, should increase the piezocatalytic performance, which is not what is observed herein. Figure 3f shows the magnetization (*M*) versus magnetic field (*H*) for all BLFO samples. One can observe that all the BLFO NPs present a weak ferromagnetic behavior, and even the pristine BFO NPs show a non‐zero remanent magnetization *M*_r_ of ≈0.019 Am^2^ Kg^−1^. 1% La‐doping (i.e., for *x* = 0.01) opens the *M‐H* hysteresis loop, as shown in the inset of Figure 3f, and enhances the *M*_r_ and the coercive field *H*_c_ to reach a maximum value of 0.029 Am^2^ Kg^−1^ and 67.8 T, respectively. Note that the BLFO *x* = 0.05 has parasitic phases that do not contribute to the magnetic response of the whole sample. Moreover, as the *M‐H* loop coincides with that of pure BFO, it strongly suggests that the spin cycloidal modulation which is known to destabilize and cause weak ferromagnetism when disturbed by doping or size effect, is not altered here. Bi_0.99_La_0.01_FeO_3_ NPs which exhibit the best piezocatalytic activity therefore show singular and different magnetic properties suggesting that a magnetic defect contributes to the magnetic response. A closer look to the core level Fe 2p_3/2_ XPS (Figure S12, Supporting Information) response shows that the difference between the spectra of La‐doped and pure BFO that a band located at 709.65 eV corresponding to Fe^2+^ elements can be evidenced. The existence of Fe^2+^ in Bi_0.99_La_0.01_FeO_3_ NPs could explain the increase of *M*_r_ by the destabilization of the spin cycloid and the increase of *H*_c_ because Fe^2+^ will increase the spin‐orbit coupling compared to Fe^3+^. It is also worth mentioning that the substitution of Fe^3+^ by Fe^2+^ provides a negatively charged defect ($Fe_{Fe}^{-}$). These defects can couple to $V_{O}^{2+}$ to form a defect dipole $Fe_{Fe}^{-}-V_{O}^{2+}$. These defect dipoles are known to impact on the ferroelectric properties,^[^ ^20^, ^38^ ^]^ by introducing internal fields (usually coined imprint fields) which we believe will contribute to better charge separation when added to the piezopotential due to ultrasound stimulus. The better piezocatalytic response of Bi_0.99_La_0.01_FeO_3_ NPs compared to *x* = 0 and *x* = 0.03 can be thus understood by the synergetic effects of Fe^2+^ and oxygen vacancy defects. While difficult to probe, especially in nanoparticles and with so tiny amount of defects, we believe that defects and associated defects such as defect dipoles play a significant role in the piezocatalytic mechanism, in addition to both charge‐screening and band structure contributions due to the presence of the piezopotential. This work therefore adds a new avenue for the model multiferroic BFO in purifying water pollutants and a designing approach toward high‐performance piezocatalysts through defect engineering.

## 3 Conclusion

In summary, we have shown that a full degradation efficiency is observed in Bi_0.99_La_0.01_FeO_3_ NPs for decomposing the Rhodamine B model pollutant under ultrasonic excitations in the dark, corresponding to the kinetic rate constant of 21 360 L mol^−1^ min^−1^. Such ultrafast degradation rate never observed in any ferroelectric materials up to now is attributed to the presence of oxygen vacancies supported by XPS, EPR, and optical measurements in the NPs, acting as charge providers, and defect dipoles when coupled with Fe^2+^‐defects, supported by XPS and magnetic measurements, which provide an internal electric field that contributes to the efficient charge separation when added to the piezopotential under the ultrasonic stimulus. Moreover, we have demonstrated that these Bi_0.99_La_0.01_FeO_3_ NPs exhibit a wide piezocatalytic versatility allowing them to piezo‐degrade a cocktail of pharmaceutical pollutants, with over 90% efficiency. These findings, which may only be the tip of the iceberg, further validate the fascinating prospect of utilizing BFO‐based ferroelectrics in wastewater treatment and open up a prodigious possibility to explore such “ferrocatalysts” in various applications including hydrogen production and CO_2_ reduction or biomedicine (e.g., sterilization, tumor therapy and biosensing). This work adds a new path for designing highly efficient piezocatalysts by using defect engineering.

## 4 Experimental Section

### Catalysts Synthesis

La‐doped BiFeO_3_ nanoparticles (Bi_1‐x_La_x_FeO_3_: *x* = 0, 0.01, 0.03, and 0.05) were synthesized using the chemical bath method.^[^ ^8^, ^27^, ^35^ ^]^ Typically, an appropriate amount of bismuth nitrate (Bi(NO_3_)_3_.5H_2_O, 98% Alfa Aesar), iron nitrate (Fe(NO_3_)_3_.9H_2_O, 98–101% Alfa Aesar) and lanthanum nitrate (La(NO_3_)_3_.6H_2_O, 99.9% Alfa Aesar) were used as precursors and were dissolved in distilled water. Next, ammonia was added to the solution as a complexant. The precursor solution was subsequently heated at 50 °C for 2 h under continuous magnetic stirring. The resulting precipitant was then collected and washed with distilled water several times and finally annealed at 500 °C for 2 h in air.

### Characterization

The crystalline structure of the nanoparticles was performed by X‐ray diffraction (XRD, Malvern PANalytical Aeris diffractometer). The unit cell parameters were obtained by the Rietveld refinement method using Jana 2006 software. Raman spectra were collected at room temperature on a LABRAM Horiba‐Jobin‐Yvon spectrometer equipped with a He‐Ne laser (633 nm) as an excitation source. The morphology and the size of the NPs were assessed by transmission electron microscopy (TEM) and high‐resolution TEM (JEOL JEM 2100) operated at 200 kV. X‐ray photoelectron spectroscopy (XPS) was conducted on a PHI 5000 VersaProbe operating with a focused monochromatized Al Kα X‐ray source. Nitrogen adsorption measurements were performed at 77 K on a Quantachrome Autosorb‐iQ Station2 instrument (Anton Paar). The samples were degassed in a vacuum at 100 °C. X‐band Electron Paramagnetic Resonance (EPR) measurements were conducted using Bruker EMX Nano Benchtop spectrometer with an integrated referencing for g‐factor calculation. A 9.64 GHz microwave frequency was applied to the samples. All EPR spectra were measured using 25 cm long spin‐free quartz tubes at room temperature with a modulation amplitude of 2 G. Magnetic measurements were carried out at 300 K using vibrating sample magnetometers (VSM 7304, Lake Shore Cryotronics). Room temperature UV–vis diffuse reflectance spectra were measured using a Perkin Elmer spectrometer (Lambda 850) equipped with a Harrick Praying MantisTM diffuse reflectance accessory. The non‐absorbing standard BaSO_4_ powder has been used as a reflectance reference.

### Piezocatalytic Measurements

Rhodamine B (RhB) was used as the target pollutant to test the piezocatalytic degradation performance under ultrasonic vibration. In a typical experiment, 10 mg of catalyst was dispersed in 10 mL of RhB solution (10^−5^ m). Subsequently, the suspension was magnetically stirred for 30 min in the dark to achieve complete adsorption‐desorption equilibrium between the catalyst and RhB molecules. An ultrasonic cleaner (100 W, 45 kHz), was used to exert the external mechanical forces on the catalysts. At regular intervals, aliquots (≈3 mL) were taken from the reactor and the dye concentration was determined by UV−vis spectroscopy (Perkin Elmer spectrometer, Lambda 850). To avoid the effect of light, the catalytic experiments were performed in the dark. The piezocatalytic degradation reaction of different antibiotic pharmaceuticals, named tetracycline (TC, Alfa Aesar), carbamazepine (CBZ, 98% Alfa Aesar), and ciprofloxacin (CIP, 98% Alfa Aesar) were also implanted. To reveal the active species participating in the piezocatalytic process, *tert*‐butyl alcohol (TBA, 99% Alfa Aesar), benzoquinone (BQ, 98% Alfa Aesar), and disodium ethylene diamine tetra‐acetate (EDTA, 99% Alfa Aesar) were introduced into the catalytic system as the scavengers of hydroxyl radicals (•OH), superoxide radicals (•O_2_^−^), and holes (h^+^), respectively at a concentration of 1 mm.

## Conflict of Interest

The authors declare no conflict of interest.

## Author Contributions

W.A. conceived the study, synthesized the materials, conducted the piezocatalytic experiments, and carried out the necessary characterizations. B.D. and J.K. supervised the work. M.O. performed TEM and HRTEM measurements. D.A. and E.E. did the XPS and EPR measurements, respectively. Magnetic measurements were carried out and analyzed by F.M. Raman spectra collected by P.G. S.G. performed the N_2_ adsorption measurements. W.A. analyzed the data and wrote the manuscript. H.M.M. and all of the authors commented on the manuscript.

## Supporting information



Supporting Information

## Data Availability

The data that support the findings of this study are available from the corresponding author upon reasonable request.
