# Supplementary material for: Enhancement of the Piezocatalytic Response of La‐Doped BiFeO3 Nanoparticles by Defects Synergy
Source: Small. 2024 Sep 30;20(50):2406425. doi: 10.1002/smll.202406425 (PMC11636164; doi:10.1002/smll.202406425)
Supplement: Supplementary file 1 — Supporting Information [file SMLL-20-2406425-s001.docx]

Supporting Information

Enhancement of the Piezocatalytic Response of La-Doped BiFeO_3_ Nanoparticles by Defects Synergy

Wafa Amdouni,^1,2^* Mojca Otoničar,^3^ David Alamarguy,^4^ Emre Erdem,^5^ Pascale Gemeiner,^1^ Frédéric Mazaleyrat,^6^ Hager Maghraoui-Meherzi,^2^ Jens Kreisel,^7^ Sebastjan Glinsek,^8^ and Brahim Dkhil^1^

^1^ Université Paris-Saclay, CentraleSupélec, Laboratoire Structures, Propriétés et Modélisation des Solides, UMR CNRS 8580, 91190 Gif-sur-Yvette, France

^2^ Université de Tunis El-Manar, Faculté des Sciences de Tunis, Laboratoire de Chimie Analytique et Électrochimie LR99ES15, Campus Universitaire de Tunis El-Manar, 2092 Tunis, Tunisie

^3^ Jožef Stefan Institute, Jamova 39, 1000, Ljubljana, Slovenia

^4^ Université Paris-Saclay, CentraleSupélec, CNRS, Laboratoire de Génie Electrique et Electronique de Paris, 91192, Gif-sur-Yvette, France

^5^ Faculty of Engineering and Natural Sciences & Center of Excellence for Functional Surfaces and Interfaces for Nano-Diagnostics (EFSUN), Sabanci University, 34956, Orhanli, Istanbul, Turkey

^6^ Université Paris-Saclay, ENS Paris-Saclay, CNRS, SATIE, 91190 Gif-sur-Yvette, France

^7^ Department of Physics and Materials Science, University of Luxembourg, L-4422 Belvaux, Luxembourg

^8^ Institute of Science and Technology, 41 rue de Brill, L-4422 Belvaux, Luxembourg

*Email: [wafa.amdouni@centralesupelec.fr](mailto:wafa.amdouni@centralesupelec.fr) or [wafa.amdouni@fst.utm.tn](mailto:wafa.amdouni@fst.utm.tn)


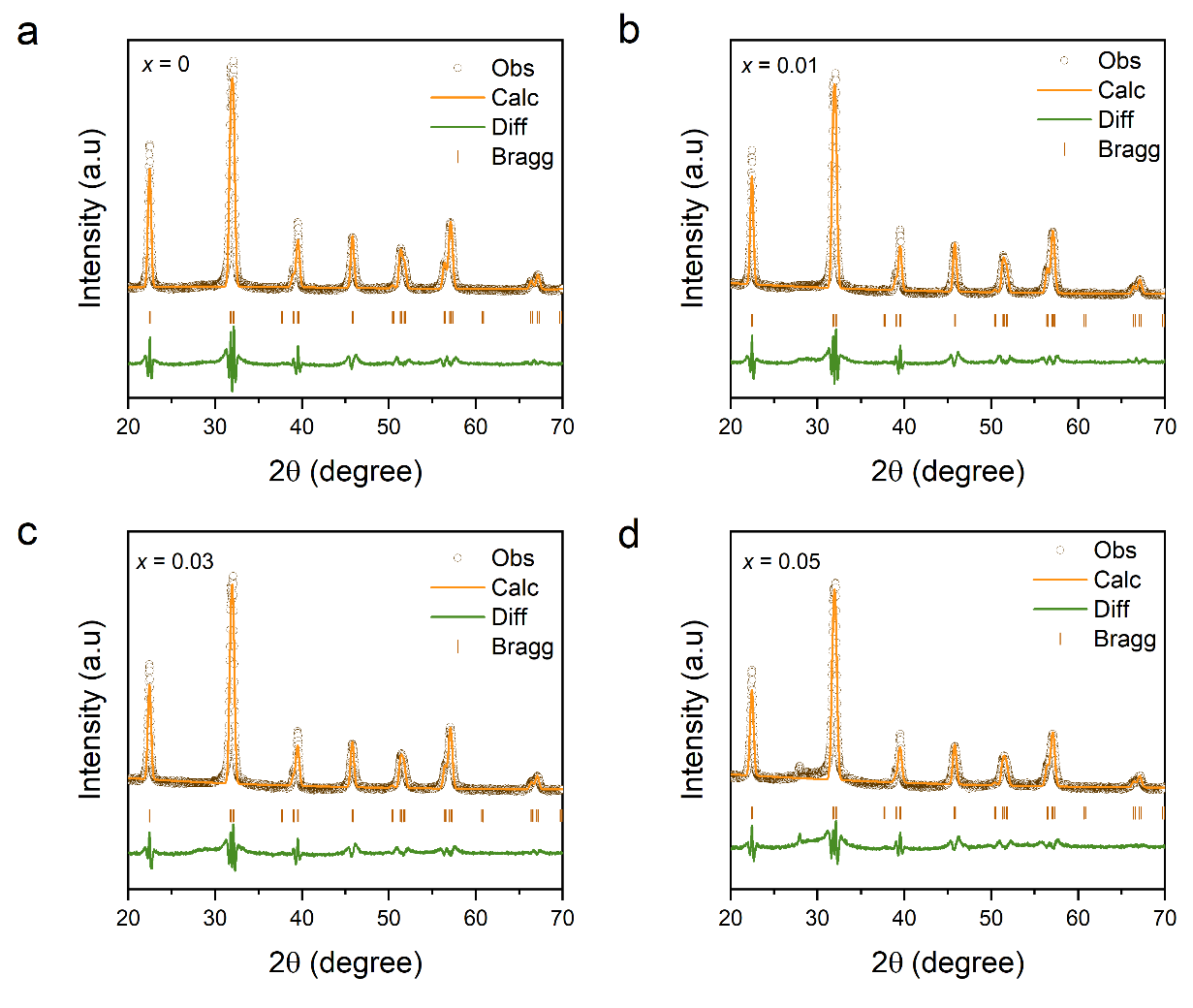


**Figure S1**. Rietveld refinement of XRD patterns of Bi_1-x_La_x_FeO_3_ nanoparticles. All these graphs include the experimental data (symbols), calculated pattern (orange line), difference plot (green line) and Bragg position (brown vertical line).


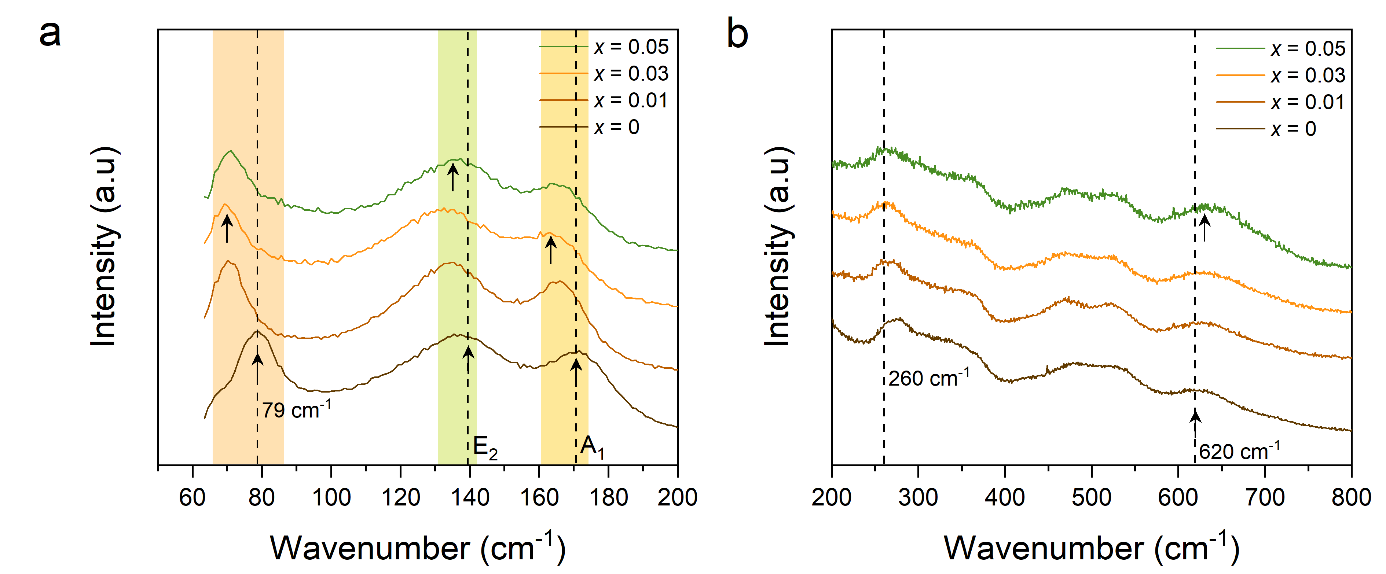


**Figure S2.** Room temperature Raman spectra of Bi_1-x_La_x_FeO_3_ nanoparticles in the spectral range: a) 50-200 cm^-1^ and b) 200-800cm^-1^. Vertical dashed lines indicate the mode reference position for pure BiFeO_3_. Arrows are a guide to the eye, indicating the evaluated position of modes with La-doping. Note that the Raman spectra are separated into two panels to facilitate comparison between samples. Note also that the change of behavior (up-shift of the 79 cm^-1^ mode) could be attributed to the presence of parasitic phases (thus less La inserted into BFO).


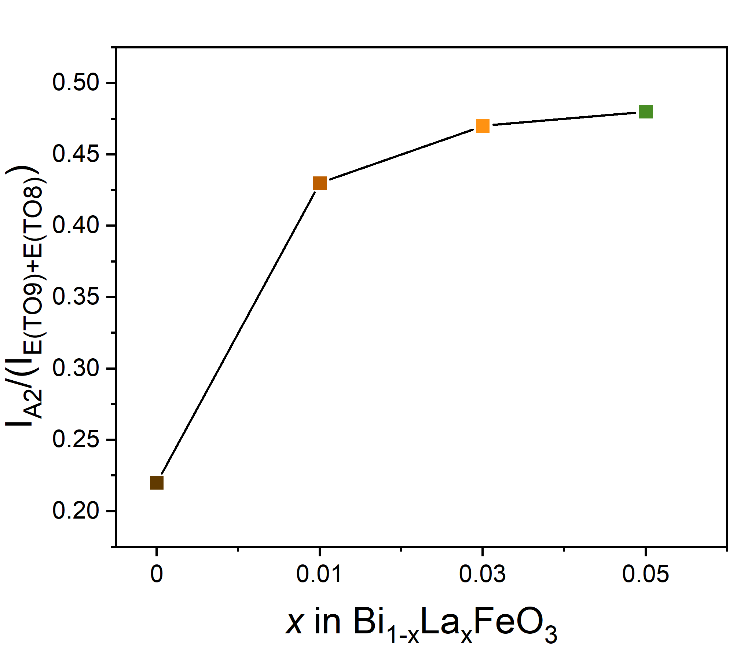


**Figure S3.** Intensity ratio of silent A_2_ longitudinal optical and E(TO9)+E(TO8) mode for Bi_1-x_La_x_FeO_3_ nanoparticles.


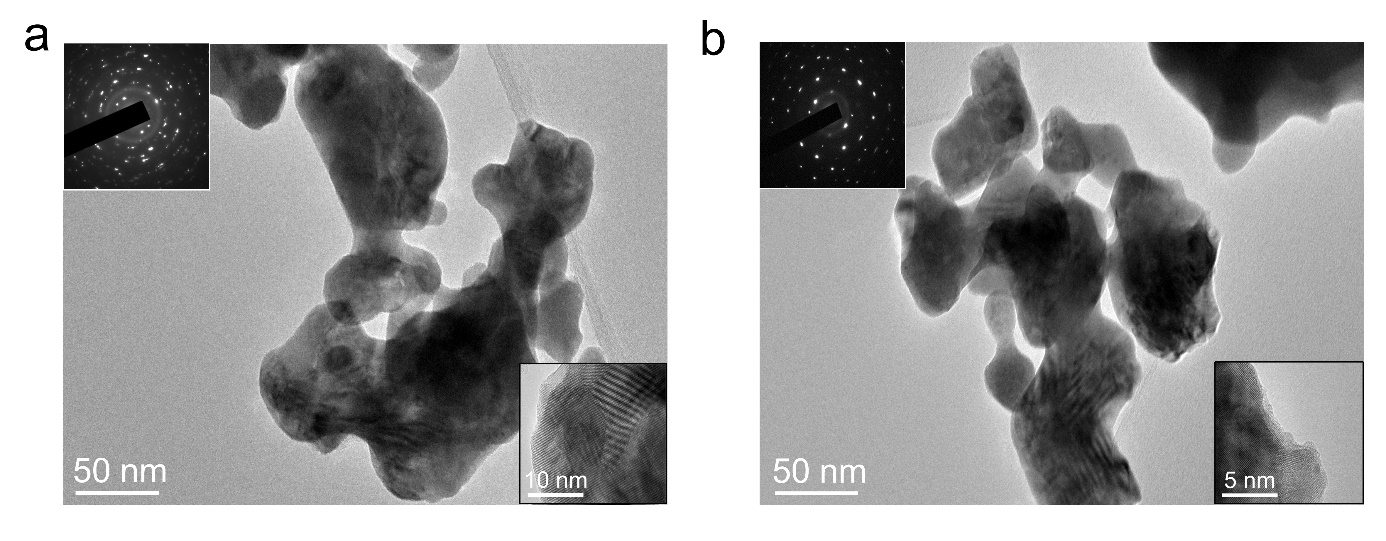


**Figure S4.** TEM images of (a) Bi_0.97_La_0.03_FeO_3_ and (b) Bi_0.95_La_0.05_FeO_3_ nanoparticles. Insets in the figures show their corresponding SAED pattern (top left) and HRTEM images (bottom right).


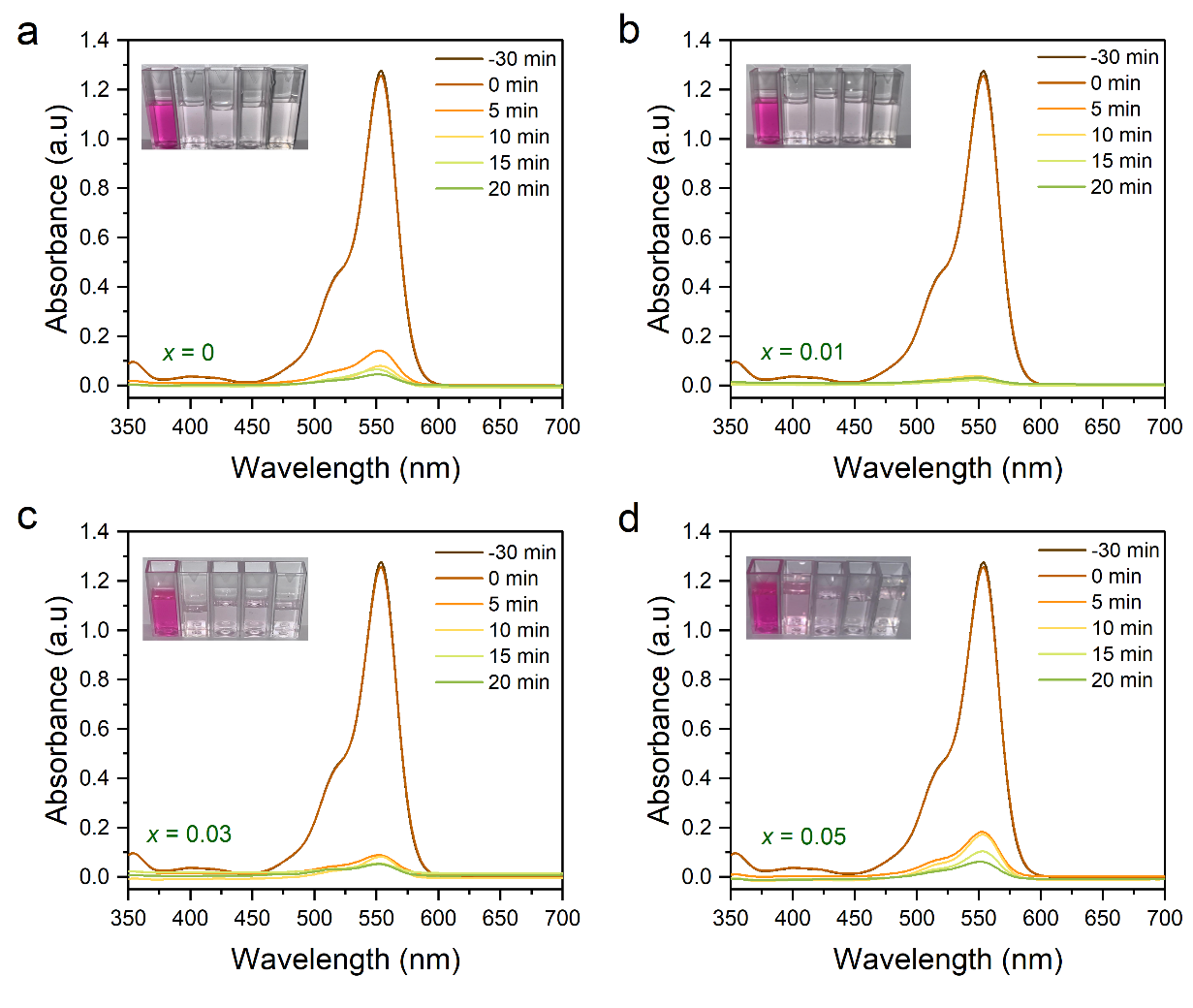


**Figure S5.** UV-vis absorption spectra for piezocatalytic degradation of RhB using Bi_1-x_La_x_FeO_3_ nanoparticles. The insets are photographs showing the piezocatalytic decomposition of the RhB solution in the presence of Bi_1-x_La_x_FeO_3_ nanoparticles before and after the piezocatalysis process. The adsorption-desorption equilibrium between the catalysts NPs and RhB is achieved within 30 min.


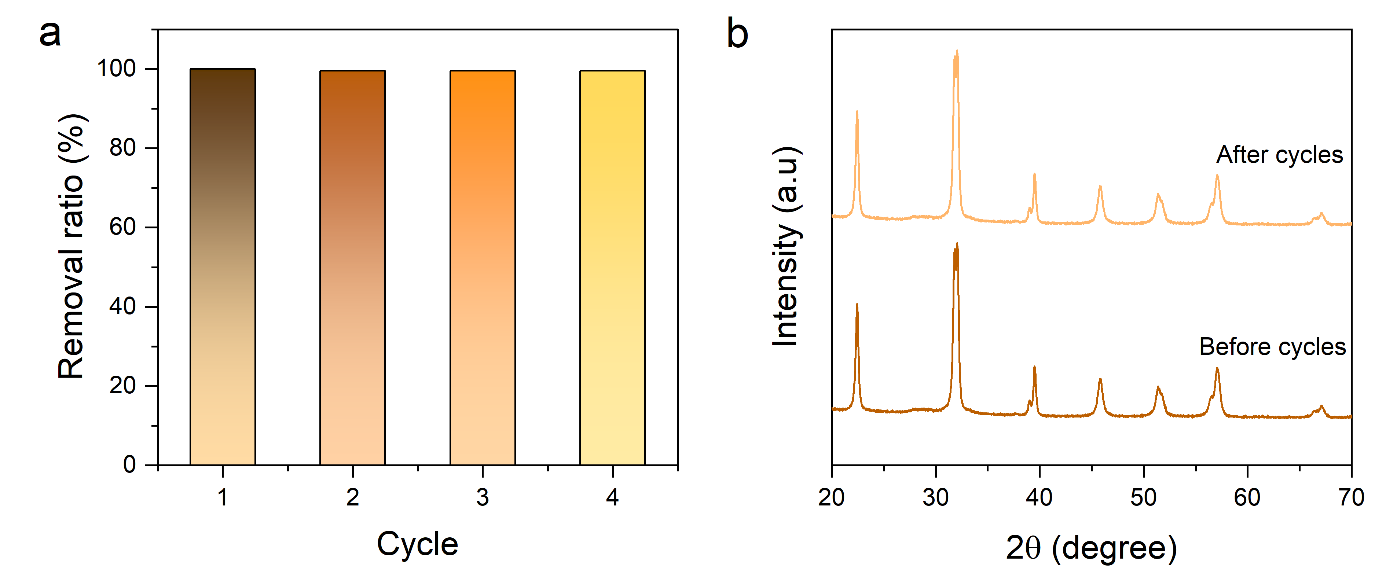


**Figure S6**. a) Removal rate of RhB using Bi_0.99_La_0.01_FeO_3_ nanoparticles for four consecutive cycles. b) XRD pattern of Bi_0.99_La_0.01_FeO_3_ before and after four cycles of piezocatalytic degradation of RhB.


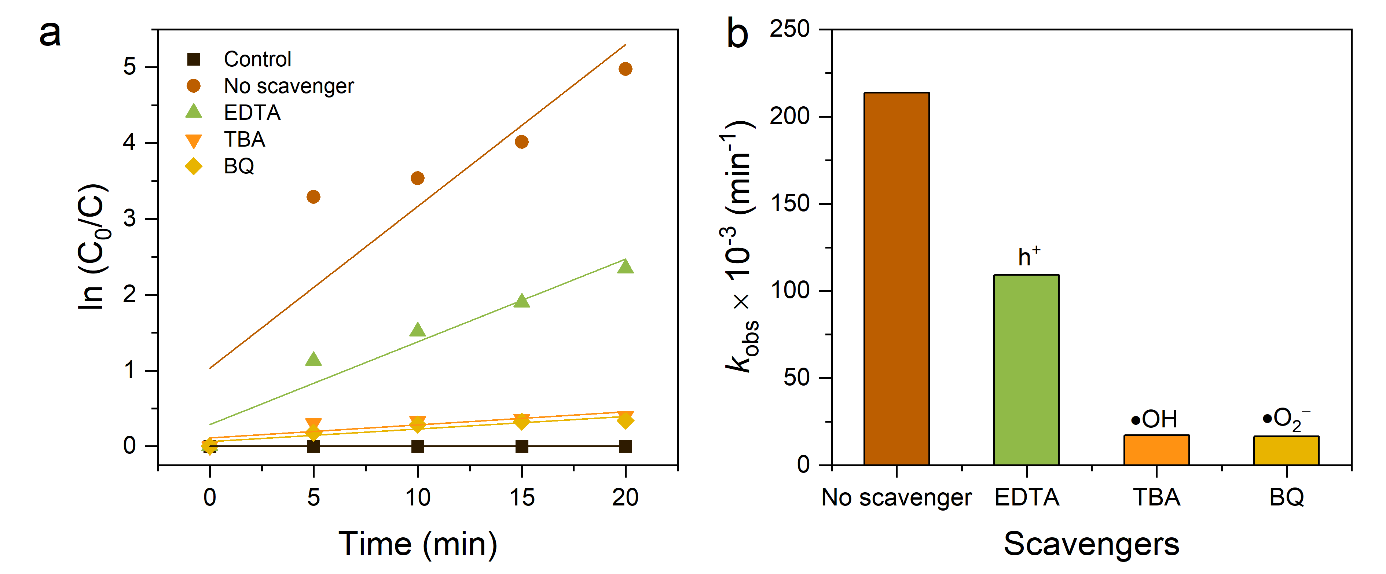


**Figure S7.** a) Pseudo-first-order kinetics fitting data for piezocatalytic degradation of RhB with and without free radical scavengers using Bi_0.99_La_0.01_FeO_3_ nanoparticles. b) The corresponding reaction k_obs_ constant.

The contribution of the reactive active species generating during the piezocatalytic process was calculated by the following Equations:

|  | $\left( \text{•}OH \right)=\frac{k_{RhB}-k_{\text{•}OH}}{k_{RhB}}\times100$ | (1) |
| --- | --- | --- |
|  | $\left( \text{•}O_{2}^{-} \right)=\frac{k_{RhB}-k_{\text{•}O_{2}^{-}}}{k_{RhB}}\times100$ | (2) |
|  | $\left( h^{+} \right)=\frac{k_{RhB}-k_{h^{+}}}{k_{RhB}}\times100$ | (3) |

where, λ is the contribution rate of the reactive species, and *k*_RhB_, *k*_•OH_, $\text{k}_{\bullet O_{2}^{-}}$ and *k*_h+_ are the pseudo-first-order rate constant of the degradation of RhB without and with TBA, BQ, and EDTA-2Na scavengers, respectively, in the presence of Bi_0.99_La_0.01_FeO_3_ nanoparticles.


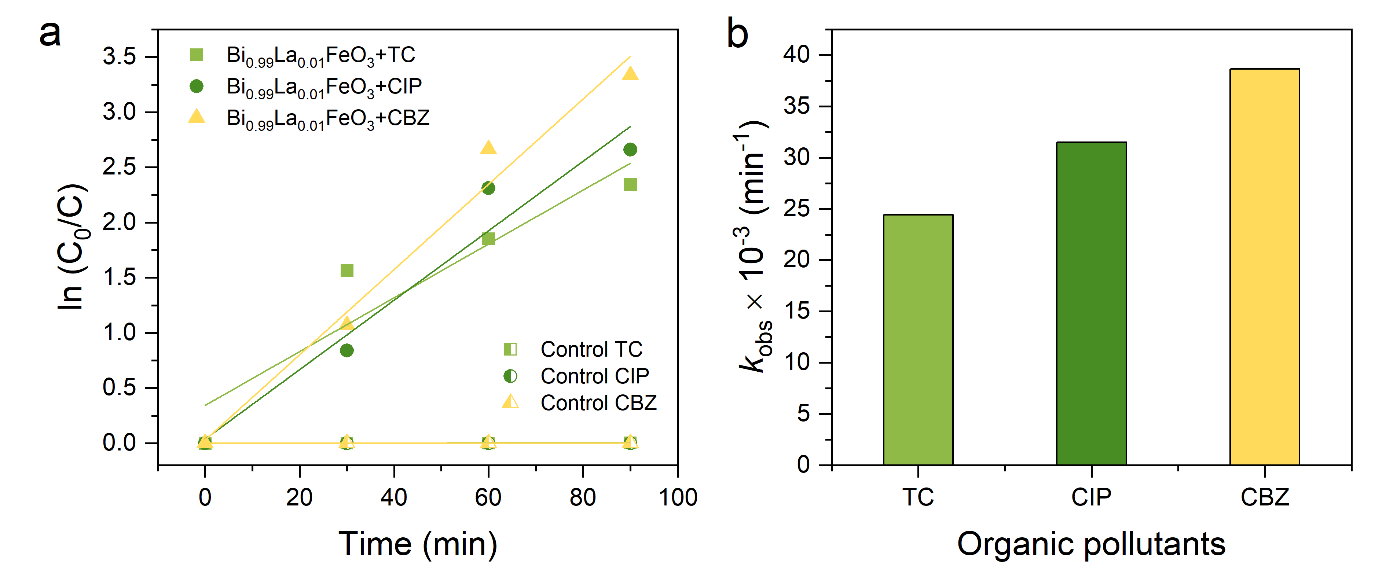


**Figure S8.** a) Pseudo-first-order kinetics fitting data for piezocatalytic degradation of TC, CIP, and CBZ using Bi_0.99_La_0.01_FeO_3_ as catalysts. b) The corresponding reaction k_obs_ constant.


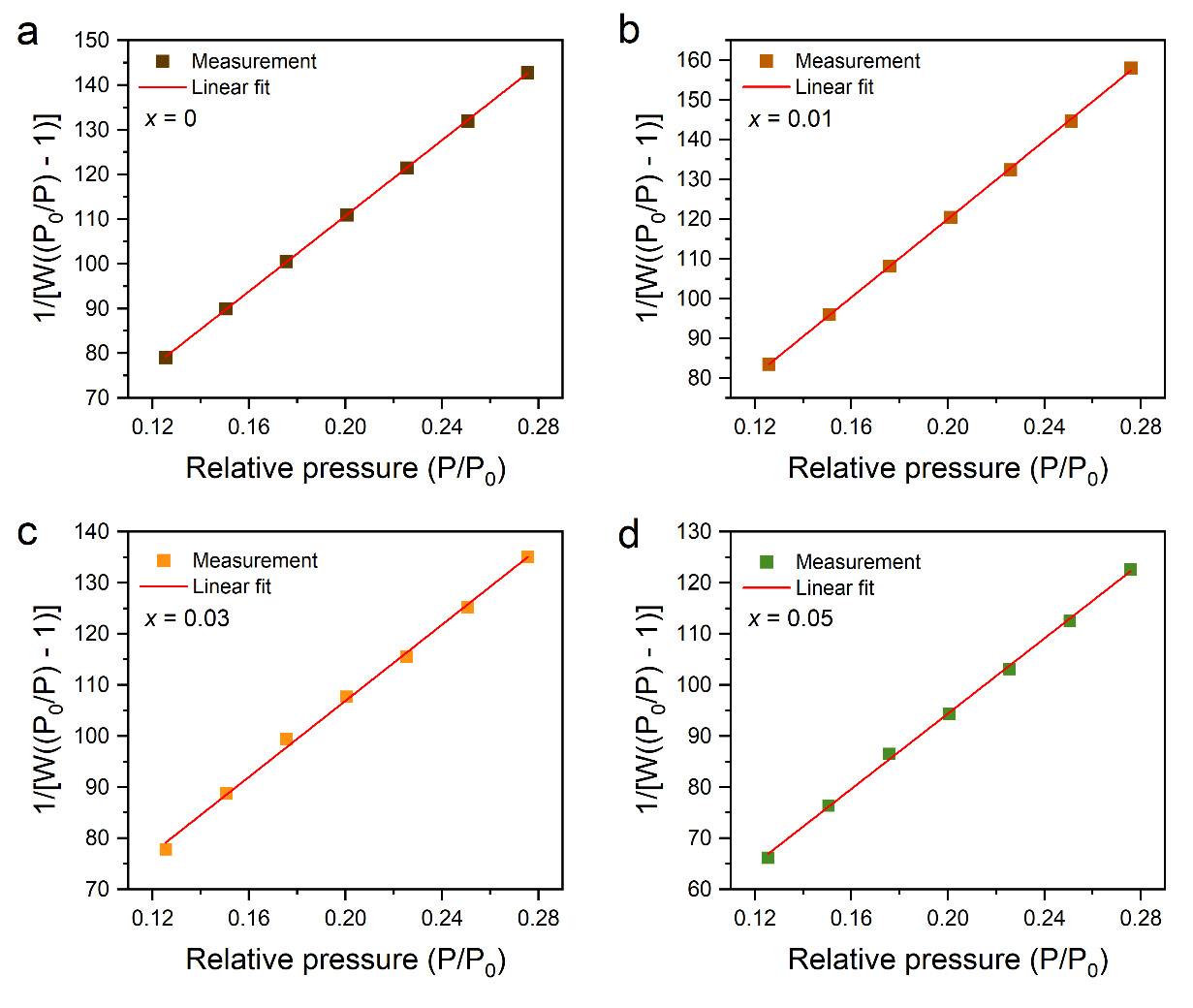


**Figure S9.** BET plots of Bi_1-x_La_x_FeO_3_ nanoparticles.


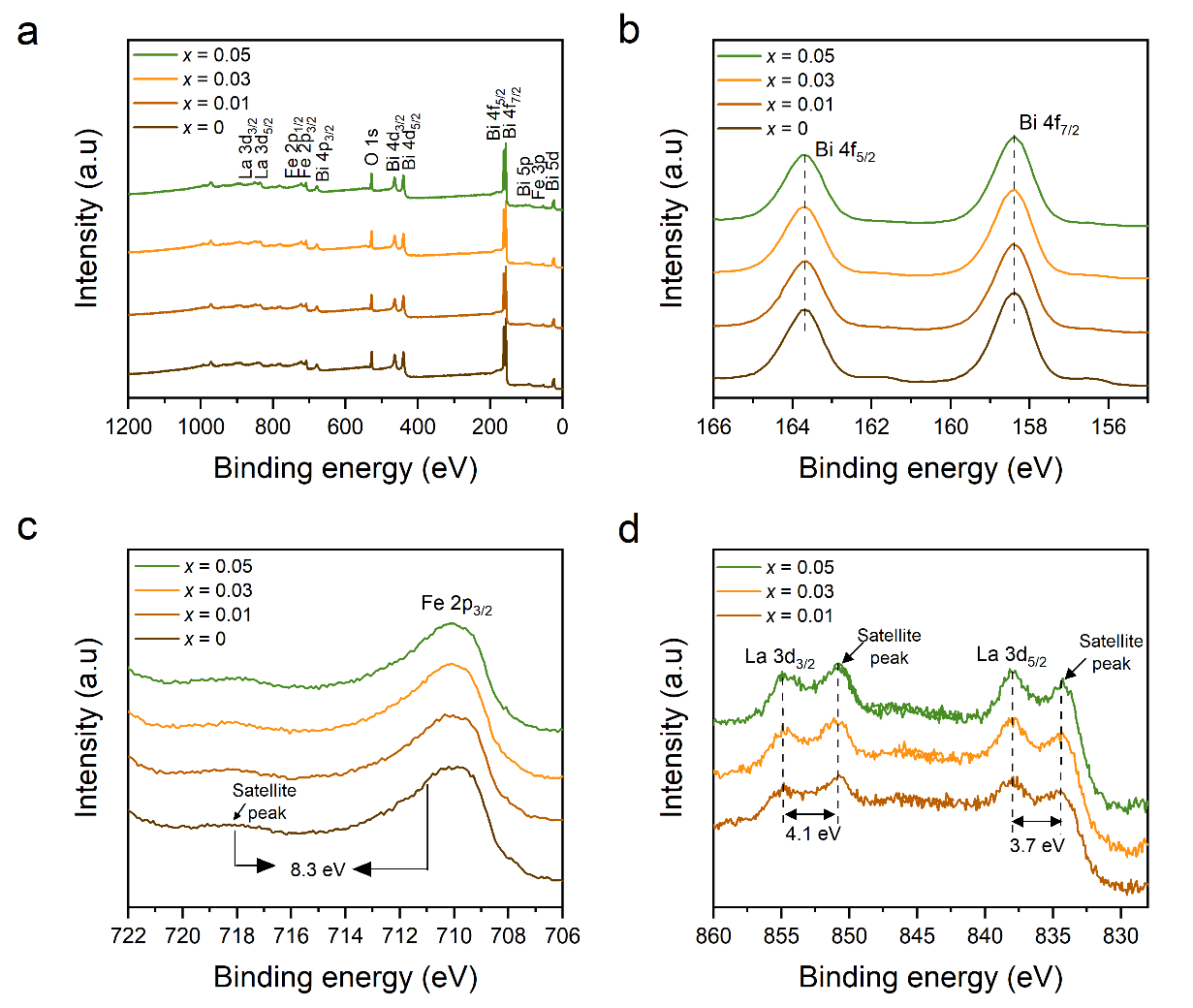


**Figure S10.** a) XPS survey spectra of Bi_1-x_La_x_FeO_3_ nanoparticles. High resolution XPS spectra of a) Bi 4f, c) Fe2p and d) La 3d, respectively.

The XPS survey spectra of Bi_1-x_La_x_FeO_3_ NPs (*x* = 0, 0.01, 0.03, 0.05) are given in Figure S10a. At first glance, it can be seen that the spectra of BLFO NPs are same in the whole scanning range. Figure S10b shows the Bi 4f core level spectra, consisting of two strong peaks that correspond to Bi-O bonds. These peaks located at ≈158.4 and ≈163.7 eV correspond to Bi 4f_7/2_ and Bi 4f_5/2_ of Bi^3+^ state, respectively.^[1]^ Moreover, two distinct peaks were observed at ≈156.4 and 161.7 eV, and which can be ascribed to the presence of metallic bismuth Bi^0^.^[2]^ In Figure S10c, the Fe 2p_3/2_ core level region of Fe 2p is represented, with their satellite peak located at ≈718.2 eV (≈8.3 eV above the main peak Fe 2p_3/2_), which confirm the presence of Fe^3+^ in all the as-synthesized NPs. However, as shown in Figure S12, we cannot exclude the presence of Fe^2+^ in the total Fe signal. The satellite peak originates from the motion of 3d electron to vacant 4s orbitales during the ejection of electron from the 2p sell, the so-called sake-up transition.^[3]^ In the La 3d spectrum (Figure S10d), two peaks centering at ≈837.5 and 854.72 eV matches Lanthanum in the La^3+^ state, confirming the successful doping of La.


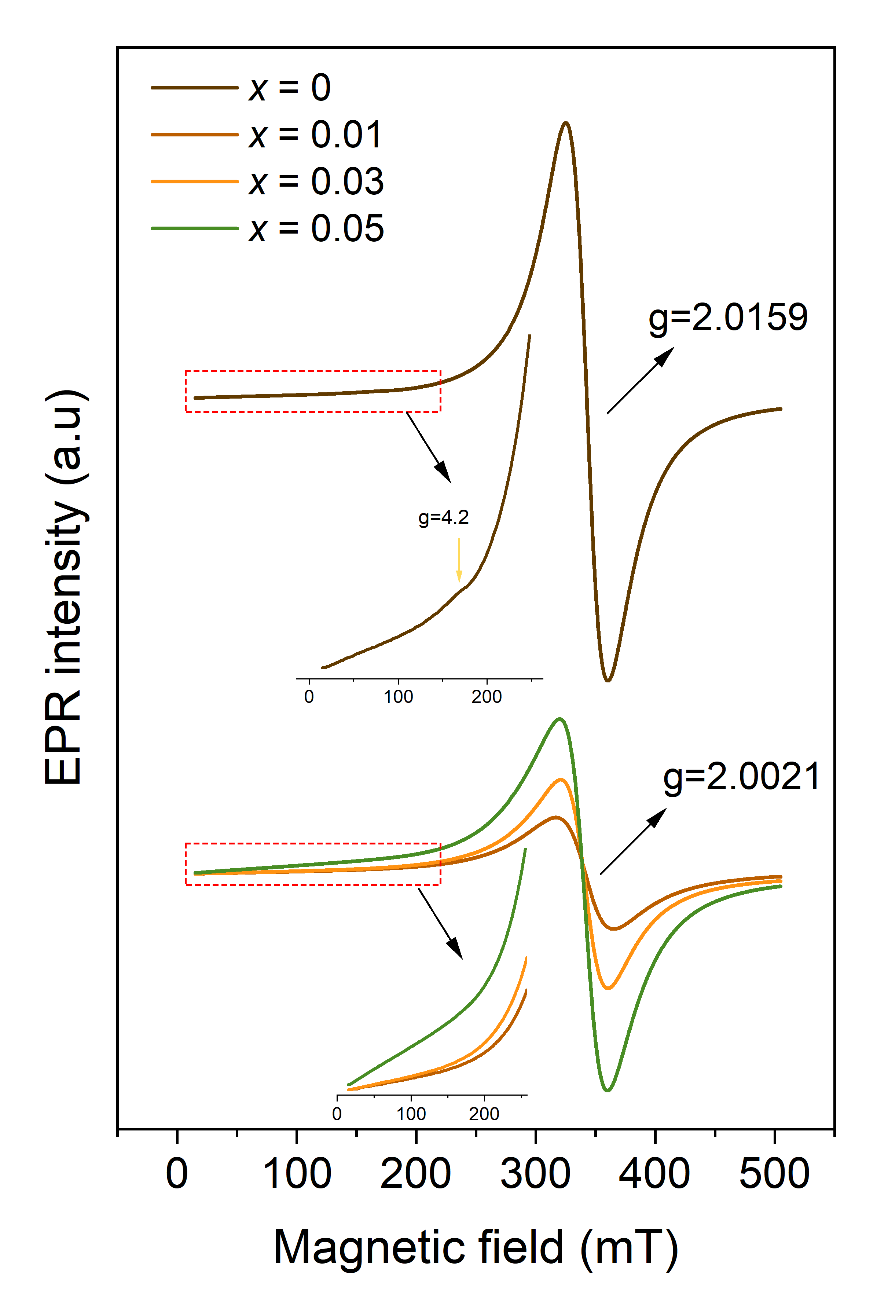


**Figure S11**. Room temperature EPR spectra of pure and various amount of Bi_1-x_La_x_FeO_3_ nanoparticles.

Figure S11: Room temperature Electron Paramagnetic Resonance (EPR) spectra of pure and various amount of La doped BFO samples. The EPR spectra of pure BiFeO_3_ (BFO) samples exhibit distinct signals indicative of both regular substitutional Fe^3+^ ions and Fe^3+^ ions coupled with oxygen vacancies. At room temperature, the EPR spectrum of pure BFO shows a prominent resonance signal at a magnetic field of approximately 350 mT, corresponding to a g-factor of 2.0159 and a peak-to-peak linewidth (ΔH_PP_) of 36 mT. This intense signal is characterized by a single Lorentzian line shape, consistent with the Fe^3+^ spin-spin exchange interaction, a well-known phenomenon that results in dipolar broadening. The presence of this large EPR signal corroborates recent findings in the literature. ^[4]^ Additionally, a weaker signal is observed at a lower magnetic field of approximately 167 mT. This signal, with a g-factor of 4.2, is significantly weaker compared to the primary Fe^3+^ spin-spin interaction signal. To better visualize this low-field profile, the corresponding region of the EPR spectrum has been magnified in an inset of Figure S11. The g=4.2 resonance is clearly discernible and is attributed to Fe^3+^ ions in close proximity to oxygen vacancies. These oxygen vacancies create localized magnetic environments that alter the EPR response of the Fe^3+^ ions, resulting in the observed g=4.2 signal. The EPR data provide compelling evidence for the presence of oxygen vacancies in the pure BFO sample. Doping BFO with La ions results in notable changes in the EPR spectra, reflecting alterations in the crystal structure and defect chemistry. The lineshape of the EPR signal transitions from a Lorentzian to a Gaussian profile, and the signal broadens, indicating a reduction in the symmetry (rhombohedral) of the BFO nanoparticles. This broadening suggests an increase in the concentration of oxygen vacancies as the La doping level increases. A significant observation is the shift in the g-factor from 2.0159 to 2.0021, indicating a weakening of the spin-orbit (S-L) coupling compared to the pure BFO sample. The free electron g-factor of 2.0023, which lacks orbital contribution, suggests that systems with similar g-factors generally have zero orbital momentum (L=0), as seen in ions such as Mn^2+^ and Fe^3+^. ^[5]^ This shift implies that oxygen vacancies in La-doped BFO are predominantly located on the surface, whereas in pure BFO, the vacancies are more uniformly distributed within the lattice. For La-doped BFO samples, the EPR signal of Fe^3+^ ions coupled with oxygen vacancies becomes overwhelmingly difficult to evidence. However, the increased baseline indicates that the inhomogeneous distribution of these defects causes the g=4.2 signal to be hidden within the baseline. This spatial distribution is attributed to the presence of non-equivalent magnetic species of defect dipoles.


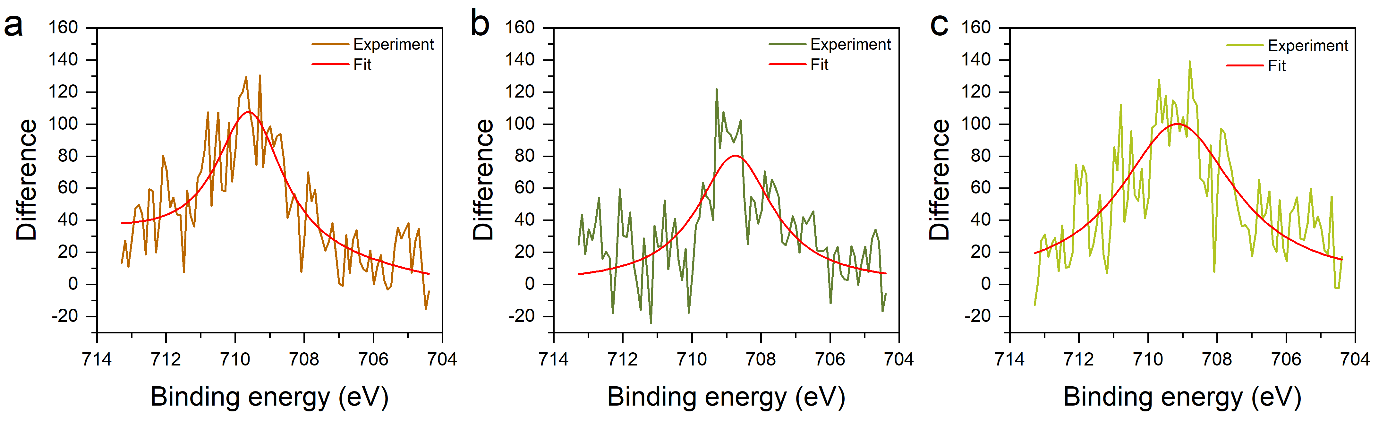


**Figure S12.** Zoom of the core level Fe 2p_3/2_ XPS for a) Bi_0.99_La_0.01_FeO_3_, b) Bi_0.97_La_0.03_FeO_3_ and (c) Bi_0.95_La_0.05_FeO_3_ nanoparticles. Note that the difference in (c) cannot be considered as it is probably altered by the presence of the parasitic phases, we evidenced in Bi_0.95_La_0.05_FeO_3_ NPs. Note also that the scale in (a) and (b) is the same, and thus the intensity difference in (a) is higher than in (b) suggesting higher amount of Fe^2+^ in Bi_0.99_La_0.01_FeO_3_, than in Bi_0.97_La_0.03_FeO_3_.


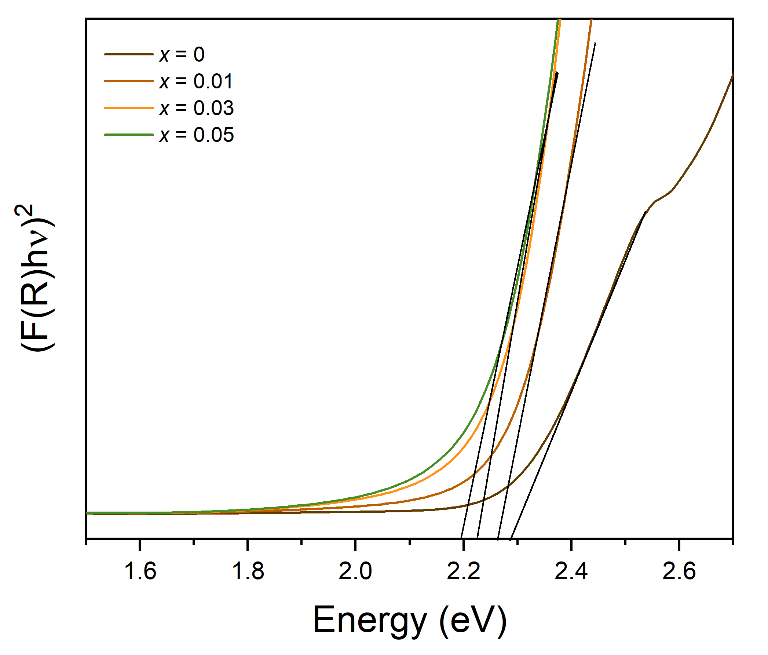


**Figure S13.** Zoom of the Tauc plots, with F(R)=(1-R)^2^/2R is the Kubelka-Munk function, R is the reflectance, and hν is the photon energy, showing the extraction of the linear region to obtain the optical bandgap for Bi_1-x_La_x_FeO_3_ nanoparticles. Note that here we extract just an effective bandgap due to the presence of an absorption band at about 2.5 eV lowering the real gap of the band structure of BFO which is higher (2.6-2.9 eV).

**Table S1.** Refined structure parameters a_hex_, c_hex_ in hexagonal representation, pseudocubic tetragonality c_c_/a_c_ calculated from corresponding lattice parameters a_c_ and c_c_, unit cell volume V_hex_ and particle size.

|  | BiFeO_3_ | Bi_0.99_La_0.01_FeO_3_ | Bi_0.97_La_0.03_FeO_3_ | Bi_0.95_La_0.05_FeO_3_ |
| --- | --- | --- | --- | --- |
| symmetry | rhombohedral | rhombohedral | rhombohedral | rhombohedral |
| space group | R3c (No. 161) | R3c (No. 161) | R3c (No. 161) | R3c (No. 161) |
| a_hex_ (Å) | 5.5804(8) | 5.5784(2) | 5.5782(6) | 5.5781(9) |
| c_hex_ (Å) | 13.8778(1) | 13.8548(0) | 13.8517(3) | 13.8504(9) |
| a_c_ (Å) | 3.9459(9) | 3.9445(3) | 3.9444(2) | 3.9443(7) |
| c_c_ (Å) | 4.0061(7) | 4 | 3.9986(5) | 3.9982(9) |
| c_c_/a_c_ | 1.0152(5) | 1.0140(6) | 1.0137(4) | 1.0136(7) |
| V_hex_ (Å^3^) | 374.27 | 373.38 | 373.27 | 373.23 |
| R_p_ | 8.69 | 5.81 | 5.69 | 6.19 |
| R_wp_ | 6.38 | 7.88 | 7.73 | 8.44 |
| GOF | 3.30 | 3.21 | 3 | 3.41 |
| GOF: Goodness of Fit | | | | |

**Table S2.** Raman modes of Bi_1-x_La_x_FeO_3_ nanoparticles (*x* = 0, 0.01, 0.03, and 0.05).

| Raman Modes | Wavenumber (cm^-1^) | | | |
| --- | --- | --- | --- | --- |
|  | BiFeO_3_ | Bi_0.99_La_0.01_FeO_3_ | Bi_0.97_La_0.03_FeO_3_ | Bi_0.95_La_0.05_FeO_3_ |
| E(TO1) | 78 | 70 | 69 | 71 |
| E(TO2) | 140 | 135 | 134 | 134 |
| A_1_(TO1) | 170 | 167 | 166 | 164 |
| E(TO3) | 221 | 220 | 218 | 219 |
| E(TO4) | 260 | 257 | 255 | 253 |
| A_1_(TO2) | 263 | 258 | 259 | 254 |
| E(TO5) | 276 | 275 | 274 | 274 |
| A_1_(TO3) | 315 | 314.8 | 315 | 315 |
| E(TO6) | 339 | 339 | 335 | 334 |
| E(TO7) | 363 | 363.7 | 363.7 | 364 |
| E(TO8) | 472 | 471 | 471 | 469 |
| E(TO9) | 503 | 498 | 499 | 497 |
| A_1_(TO4) | 533 | 528 | 526 | 527 |

**Table S3.** Comparison of piezocatalytic performance of La-doped BiFeO_3_ for RhB degradation with other reported piezocatalysts.

| Piezocatalysts^a^ | Energy source | Organic pollutant | Organic pollutants concentration | Catalyst dosage (g L^-1^) | Time (min) | *k*_obs_ constant (×10^-3^ min^-1^) | Ref |
| --- | --- | --- | --- | --- | --- | --- | --- |
| BaTiO_3_ (BTO) | 100 W, 50 kHz | Rhodamine B | 10 mg L^-1^ | 1 | 70 | 25.3 | ^[6]^ |
| Ba_0.75_Sr_0.25_TiO_3_ (BSTO) | 40 kHz | Rhodamine B | 10 mg L^-1^ | 0.2 | 120 | 24.5 | ^[7]^ |
| BaTiO_3_/C | 150 W, 40kHz | Rhodamine B | 5 mg L^-1^ | 1 | 40 | 49.01 | ^[8]^ |
| Bi_4_Ti_3_O_12_ (BIT) | 120 W, 40 kHz | Rhodamine B | 5 mg L^-1^ | 1 | 60 | 57 | ^[9]^ |
| ZnO/BTO | 120 W, 40 kHz | Rhodamine B | 10 mg L^-1^ | 1 | 90 | 15.3 | ^[10]^ |
| BNBT | 100 W, 40 kHz | Rhodamine B | 10 mg L^-1^ | 1 | 150 | ~12 | ^[11]^ |
| LNKN | - | Rhodamine B | 5 mg L^-1^ | 4 | 100 | 25.16 | ^[12]^ |
| K_0.5_Na_0.5_NbO_3_ (KNN) | 180 W, 40 kHz | Rhodamine B | 5 mg L^-1^ | 4 | 160 | 19.8 | ^[13]^ |
| Na_0.5_Bi_2.5_Nb_2_O_9_ | 180 W, 40 kHz | Rhodamine B | 20 mg L^-1^ | 1 | 120 | 1.6 | ^[14]^ |
| NBT-BNT@Ag | 200 W, 40 kHz | Rhodamine B | 10 mg L^-1^ | 1 | 60 | 40 | ^[15]^ |
| BTO@ReS_2_ | 100 W, 40 kHz | Rhodamine B | 10 mg L^-1^ | 0.4 | 25 | ~10 | ^[16]^ |
| Sm-PMN-PT | 150 W, 40kHz | Rhodamine B | 5 mg L^-1^ | 1 | 40 | 73 | ^[17]^ |
| BiFeO_3_ (BFO) | 100 W, 45 kHz | Rhodamine B | 10^-5^ M | 1 | 30 | 138.1 | ^[18]^ |
| Bi_0.99_La_0.01_FeO_3_ | 100 W, 45 kHz | Rhodamine B | 10^-5^ M | 1 | 20 | 213.6 | This work |
| Ag/LN-PVDF | 120 W, 40 kHz | Tetracycline | 0.2 mM | - | 120 | 8.87 | ^[19]^ |
| BFO | 100 W, 45 kHz | Tetracycline | 10^-5^ M | 1 | 120 | 18.2 | ^[18]^ |
| Bi_0.99_La_0.01_FeO_3_ | 100 W, 45 kHz | Tetracycline | 10^-5^ M | 1 | 90 | 24.4 | This work |
| Bi_4_Ti_3_O_12_ | 40 kHz | Ciprofloxacin | 20 mg L^-1^ | 10 | 90 | ~8 | ^[20]^ |
| Bi_0.99_La_0.01_FeO_3_ | 100 W, 45 kHz | Ciprofloxacin | 10^-5^ M | 1 | 90 | 31.5 | This work |
| BFO | 100 W, 45 kHz | Carbamazepine | 10^-5^ M | 1 | 120 | 17.6 | ^[18]^ |
| Bi_0.99_La_0.01_FeO_3_ | 100 W, 45 kHz | Carbamazepine | 10^-5^ M | 1 | 90 | 38.65 | This work |

^a^ BNBT = 0.93(Bi_0.5_Na_0.5_)TiO_3_-0.07BaTiO_3_; LNKN = 0.94Na_0.5_K_0.5_NbO_3_-0.06LiNbO_3_; NBT-BNT@Ag = 0.95(Na_0.5_Bi_0.5_)TiO_3_-0.05Ba (Ti_0.5_Ni_0.5_)O_3-δ_@Ag;

Sm-PMN-PT = Sm-doped 0.68Pb(Mg_1/3_Nb_2/3_)- 0.32PbTiO_3_; Ag/LN-PVDF = Ag/LiNbO_3_-PVDF.

References

[1] H. You, Z. Wu, L. Zhang, Y. Ying, Y. Liu, L. Fei, X. Chen, Y. Jia, Y. Wang, F. Wang, S. Ju, J. Qiao, C.-H. Lam, H. Huang, *Angew. Chem. Int. Ed* **2019**, 58, 11779.

[2] A. Queraltó, E. György, R. Ivan, Á. Pérez del Pino, R. Frohnhoven, S. Mathur, *Crystals* **2020**, 10, 271.

[3] L. Yin, I. Adler, T. Tsang, L. J. Matienzo, S. O. Grim, *Chem. Phys. Lett* **1974**, 24, 81.

[4] M. Čebela, B. Janković, R. Hercigonja, M. J. Lukić, Z. Dohčević-Mitrović, D. Milivojević, B. Matović, *Process. Appl. Ceram* **2016**, 10, 201.

[5] J. A. Weil, J. R. Bolton, in *Electron Paramagnetic Resonance*, 2006.

[6] W. Penglei, L. Xinyong, F. Shiying, C. Xin;, Q. Meichun, L. Dan, M. O. Tadé, S. Liu, *Appl. Catal. B: Environ* **2020**, 279, 119340.

[7] P. T. Thuy Phuong, Y. Zhang, N. Gathercole, H. Khanbareh, N. P. Hoang Duy, X. Zhou, D. Zhang, K. Zhou, S. Dunn, C. Bowen, *iScience* **2020**, 23, 101095.

[8] L. Chen, Y. Jia, J. Zhao, J. Ma, Z. Wu, G. Yuan, X. Cui, *J. Colloid. Interface. Sci* **2021**, 586, 758.

[9] Q. Tang, J. Wu, X.-Z. Chen, R. Sanchis-Gual, A. Veciana, C. Franco, D. Kim, I. Surin, J. Pérez-Ramírez, M. Mattera, A. Terzopoulou, N. Qin, M. Vukomanovic, B. J. Nelson, J. Puigmartí-Luis, S. Pané, *Nano Energy* **2023**, 108, 108202.

[10] X. Zhou, S. Wu, C. Li, F. Yan, H. Bai, B. Shen, H. Zeng, J. Zhai, *Nano Energy* **2019**, 66, 104127.

[11] D. Liu, Y. Song, Z. Xin, G. Liu, C. Jin, F. Shan, *Nano Energy* **2019**, 65, 104024.

[12] A. Zhang, Z. Liu, B. Xie, J. Lu, K. Guo, S. Ke, L. Shu, H. Fan, *Appl. Catal. B: Environ* **2020**, 279, 119353.

[13] A. Zhang, Z. Liu, X. Geng, W. Song, J. Lu, B. Xie, S. Ke, L. Shu, *Ceram. Int* **2019**, 45, 22486.

[14] L. Lu, N. Liang, H. Sun, Q. Zhang, X. Hao, *J. Materiomics* **2022**, 8, 47.

[15] H. Xiao, W. Dong, Q. Zhao, F. Wang, Y. Guo, *J. Hazard. Mater* **2021**, 416, 125808.

[16] W. Liu, P. Wang, Y. Ao, J. Chen, X. Gao, B. Jia, T. Ma, *Adv. Mater* **2022**, 34, e2202508.

[17] X. Liu, M. Wang, Y. Zhou, T. Li, H. Duan, J. Li, L. Wang, Y. Li, S. Yang, J. Wu, C. Wang, X. Feng, F. Li, *Small* **2023**, 19, 2303129.

[18] W. Amdouni, M. Fricaudet, M. Otonicar, V. Garcia, S. Fusil, J. Kreisel, H. Maghraoui-Meherzi, B. Dkhil, *Adv. Mater* **2023**, 35, 2301841.

[19] G. Singh, M. Sharma, R. Vaish, *ACS Appl. Mater. Interfaces* **2021**, 13, 22914.

[20] C. Meng, J. Peng, L. Wang, H. Han, K. Yang, D. You, *Catalysts* **2023**, 13, 621.
